# The draft genomes of *Elizabethkingia anophelis* of equine origin are genetically similar to three isolates from human clinical specimens

**DOI:** 10.1371/journal.pone.0200731

**Published:** 2018-07-19

**Authors:** William L. Johnson, Akhilesh Ramachandran, Nathanial J. Torres, Ainsley C. Nicholson, Anne M. Whitney, Melissa Bell, Aaron Villarma, Ben W. Humrighouse, Mili Sheth, Scot E. Dowd, John R. McQuiston, John E. Gustafson

**Affiliations:** 1 Department of Biochemistry and Molecular Biology, Oklahoma State University, Stillwater, Oklahoma, United States of America; 2 Oklahoma Animal Disease Diagnostic Laboratory, Center for Veterinary Health Sciences, Oklahoma State University, Stillwater, Oklahoma, United States of America; 3 Special Bacteriology Reference Laboratory, Bacterial Special Pathogens Branch, Division of High-Consequence Pathogens and Pathology, Centers for Disease Control and Prevention, Atlanta, Georgia, United States of America; 4 Division of Scientific Resources, Centers for Disease Control and Prevention, Atlanta, Georgia, United States of America; 5 Molecular Research DNA Laboratory, Shallowater, Texas, United States of America; Seconda Universita degli Studi di Napoli, ITALY

## Abstract

We report the isolation and characterization of two *Elizabethkingia anophelis* strains (OSUVM-1 and OSUVM-2) isolated from sources associated with horses in Oklahoma. Both strains appeared susceptible to fluoroquinolones and demonstrated high MICs to all cell wall active antimicrobials including vancomycin, along with aminoglycosides, fusidic acid, chloramphenicol, and tetracycline. Typical of the *Elizabethkingia*, both draft genomes contained multiple copies of β-lactamase genes as well as genes predicted to function in antimicrobial efflux. Phylogenetic analysis of the draft genomes revealed that OSUVM-1 and OSUVM-2 differ by only 6 SNPs and are in a clade with 3 strains of *Elizabethkingia anophelis* that were responsible for human infections. These findings therefore raise the possibility that *Elizabethkingia* might have the potential to move between humans and animals in a manner similar to known zoonotic pathogens.

## Introduction

Organisms from the *Elizabethkingia* genus are ubiquitous and have been isolated from arthropods [1–5], lizards [6], fish [7], frogs [8], corn [9], hospital sinks and water spigots [10, 11], and the Mir space station [12]. Some *Elizabethkingia* spp. are considered opportunistic pathogens that can cause serious infections such as meningitis and bacteremia, primarily in neonates or immunocompromised individuals. In general, *Elizabethkingia* infections are associated with high mortality rates [13, 14], likely due in part to the intrinsic antibiotic resistance phenotype expressed by these organisms, with the majority of isolates showing resistance to broad spectrum β-lactams, tetracyclines, and aminoglycosides, both *in vivo* and *in vitro*, while more variability is found in resistance to vancomycin and ciprofloxacin [8, 15–42]. This variability in vancomycin susceptibility is of interest as there appear to be discrepancies between laboratory reports for a variety of *Elizabethkingia* strains which were not susceptible to vancomycin *in vitro* based on CLSI standard antimicrobial susceptibility testing methods [19, 39], and clinical reports suggesting that vancomycin exhibits *in vivo* therapeutic efficacy [19, 20, 22, 26, 32, 35, 37, 43–45]. Recently, an unprecedented *Elizabethkingia anophelis* outbreak occurred in Wisconsin, Michigan, and Illinois, with 65 confirmed cases and 20 deaths reported [46, 47]. This outbreak is particularly notable because in addition to the high case count, this outbreak was primarily community-associated rather than healthcare-associated, and to date, no reservoir for this outbreak has been identified. *E*. *anophelis* is also the etiologic agent of disease in healthcare associated outbreaks that have occurred in Illinois [48], the Central African Republic [49], Hong Kong [14], Taiwan [25], Singapore [36], and other isolated cases [23, 28, 33, 40, 50, 51].

It has been well documented that both food and companion animals may serve as reservoirs for antibiotic-resistant bacterial pathogens [52–60]. The findings of *Elizabethkingia meningoseptica* isolated from a dog suffering from bacteremia [60] and contagious *Elizabethkingia miricola* among farmed frogs [8] suggest that farm and/or companion animals may also act as reservoirs for *Elizabethkingia* with the potential to cause human disease.

We report here the draft genomes and antibiotic susceptibility profiles of two *E*. *anophelis* strains isolated from horses. Whole genome sequence analysis suggests that these two strains are clonal and closely related to certain human clinical *E*. *anophelis* isolates.

## Materials and methods

### Strains and growth conditions

Strains OSUVM-1 and OSUVM-2 were isolated in 2016 from diagnostic specimens associated with horses in Oklahoma that were submitted to the Oklahoma Animal Disease Diagnostic Laboratory. OSUVM-1 was cultured from a swab taken from an endoscope used at an equine hospital; and OSUVM-2 was isolated from a guttural pouch aspirate obtained from a 9-year-old intact female quarter horse that presented to Boren Veterinary Medical Teaching Hospital (BVMTH) with a previous history of strangles. In addition to OSUVM-1 and OSUVM-2, *Pseudomonas aeruginosa*, *Stenotrophomonas maltophilia* and *Chryseobacterium* spp. like bacteria were isolated from both specimens. All bacterial isolates were identified using MALDI-TOF MS. Working stocks of the *Elizabethkingia* isolates OSUVM-1 and OSUVM-2 were prepared from pure cultures grown on heart infusion agar (Remel, San Diego, CA, USA) supplemented with 5% defibrinated rabbit blood (Hemostat Laboratories, Dixon, CA, USA) that were incubated overnight at 37°C and subsequently stored at 4°C. Working cultures of each strain were prepared by inoculating a single colony into 3 ml of heart infusion (HIB) or Mueller Hinton broth (MHB) (Becton Dickinson and Company, Cockeysville, MD, USA) and incubated overnight (37°C, 200 rpm).

### Isolate identification using MALDI-TOF mass spectrometry

For bacterial identification, fresh colonies grown on tryptic soy agar containing 5% sheep blood (Fisher Scientific, Hampton, NH, USA) were applied to a spot on the MALDI-TOF MS target plate and overlaid with freshly made matrix solution (Bruker Daltonics, Billerica, MA USA) containing 70% formic acid (Sigma-Aldrich, St Louis, MO, USA) and α-cyano-4-hydroxycinnamic acid following the manufacturer’s recommendations. Bacterial identification was carried out using a Microflex LT MALDI-TOF mass spectrometer (Bruker Daltonics) using default settings. Bacterial peptide spectra were collected using FlexControl software (version 3.4, Bruker Daltonics) in positive linear mode with a mass range from 2 to 20 kDa and a laser frequency of 60 Hz (IS1–20 kV; IS2–18 kV; lens—6 kV; extraction delay time of 100 ns) in automatic mode by accumulating a maximum of 240 profiles (40 laser shots from six different positions of the target spot). Microbial peptide mass spectra were then analyzed using the Biotyper RTC software version 3.1 using the default settings and database version 4.0.0.1 (Bruker Daltonics). Both OSUVM-1 and OSUVM-2 were identified by MALDI-TOF MS as *E*. *meningoseptica*. This is consistent with the known insufficiency of MALDI-TOF MS default databases to correctly identify certain *Flavobacteriacae*, including species belonging to the *Chryseobacterium* and *Elizabethkingia* genera [61–63].

### Genome sequencing, assembly, annotation, and phylogenetic analysis

Genomic DNA was isolated from 3 ml overnight cultures of OSUVM-1 and OSUVM-2 grown in HIB as described above using Qiagen Genomic-tip 100/g columns (Qiagen, Germantown, MD, USA) following the manufacturer’s protocol. The resulting DNA samples were sent to Molecular Research LP (Shallowater, TX, USA) where library preparation was performed using the Nextera DNA sample preparation kit (Illumina Inc., San Diego, CA, USA). Genomic DNA was then sequenced using PacBio SMRT sequencing and Illumina MiSeq systems and assembled using SeqMan NGen® version 12.0 (DNASTAR, Madison, WI, USA) with paired end sequencing parameters on the default settings. The resulting assemblies were annotated using the Rapid Annotations Using Subsystems Technology (RAST) server [64–66] and the Prokaryote Genome Annotation Pipeline [67]. Both genomes were further analyzed using the nucleotide and protein Basic Local Alignment Search Tool (BLAST) [68, 69]. The draft genome sequences can be found under bioproject PRJNA397081. OSUVM-1 and OSUVM-2 are represented by biosamples SAMN08100548 and SAMN08100549 and nucleotide accession numbers PJMA00000000 and PJLZ00000000, respectively.

The OSUVM-1 and OSUVM-2 genomes were shared with the Special Bacteriology Reference Laboratory (SBRL) at CDC, where they were compared to the genomes of *E*. *anophelis* isolates derived from human clinical specimens which were obtained after the 2016 Wisconsin *Elizabethkingia* outbreak [30] in response to a general request from CDC to the various state public health departments for all *Elizabethkingia* isolates, which have been sequenced as a part of a larger project. Three isolates were found to be closely related to OSUVM-1 and OSUVM-2. These genomes had been sequenced from cultures grown at 35°C on heart infusion agar (Difco) supplemented with 5% rabbit blood (Hemostat Laboratories). DNA was extracted using the Zymo ZR Fungal/Bacterial DNA Microprep kit (Zymo Research, Irvine, CA; strain 16–293), or the MasterPure™ Complete DNA and RNA Purification Kit (Epicentre, Madison, WI; strains 16–487 and 17–001), according to the manufacturer’s instructions. Libraries were prepared using the NEBNext Ultra DNA library prep kit (New England Biolabs, Inc., Ipswich, MA, USA), then sequencing was done with an Illumina MiSeq instrument using a 2x250 paired-end protocol as described previously [70]. The *de Bruijn* graph *de novo* assembler in CLC Genomics Workbench version 9.0. (CLCbio, Aarhus, Denmark) was used on reads trimmed with a quality limit of 0.02 to produce draft genomes. Ambiguous nucleotides (N’s) in the resulting contigs were resolved using read alignments, and contigs were split wherever N’s could not be resolved. The accession numbers of these strains are NWMM00000000, NWMI00000000, and NWMH00000000. Genomes were aligned and single nucleotide polymorphism (SNP) trees produced using HarvestTools [71], and exported Newick files were edited using MEGA v6 [72].

### Antibiotic susceptibility testing

Minimum inhibitory concentrations (MIC) of antibiotics were determined using either standard CLSI protocols [73] for clindamycin, vancomycin, and fusidic acid, or the Sensititre automated system (Thermo Scientific, Waltham, MA, USA) following the manufacturer’s protocol for equine samples.

## Results and discussion

### Sequencing and mass spectrometry analysis

The assembly of OSUVM-1 sequence data produced 7 contigs and a genome of 4,153,767 bp (%GC = 35.5). OSUVM-1 contained 3,850 putative coding sequences (CDS), of which 3,777 were protein CDS. RAST annotation assigned function to 2,421 (64%) predicted protein CDS and identified 75 rRNA and tRNA CDS.

OSUVM-2 sequences were assembled into 10 contigs to produce a genome of 4,109,384 bp (%GC = 35.5). OSUVM-2 contained 3814 CDS, of which 3,750 were protein CDS. RAST annotation assigned function to 2,404 (64%) predicted protein CDS and identified 64 rRNA and tRNA CDS.

Bacterial identification using MALDI-TOF indicated that both OSUVM-1 and OSUVM-2 were members of the *Elizabethkingia* genus. The *Elizabethkingia* are nonmotile [42] and RAST analysis of the draft genomes of OSUVM-1 and OSUVM-2 revealed no features supporting motility and chemotaxis (S1 Table). The subsystem feature count in both strains were identical for 16 of 25 subsystems identified in the draft genomes (S1 Table). The two draft genomes differed in the feature count of the following subsystems: cell wall and capsule; virulence, disease, and defense; miscellaneous; membrane transport; iron acquisition and metabolism; protein metabolism; stress response; metabolism of aromatic compounds; and phages, prophages, and transposable elements (S1 Table). This last finding is consistent with our expectation that the loci carried by mobile genetic elements will be better represented in a complete genome than a draft genome, since a draft genome will contain a single copy of a transposon sequence (with coverage levels scaled to the number of copies of the transposon in the genome) while a complete genome will allow each gene in multiple copies to be identified.

### Core genome and phylogenetic analysis

**Fig 1:**
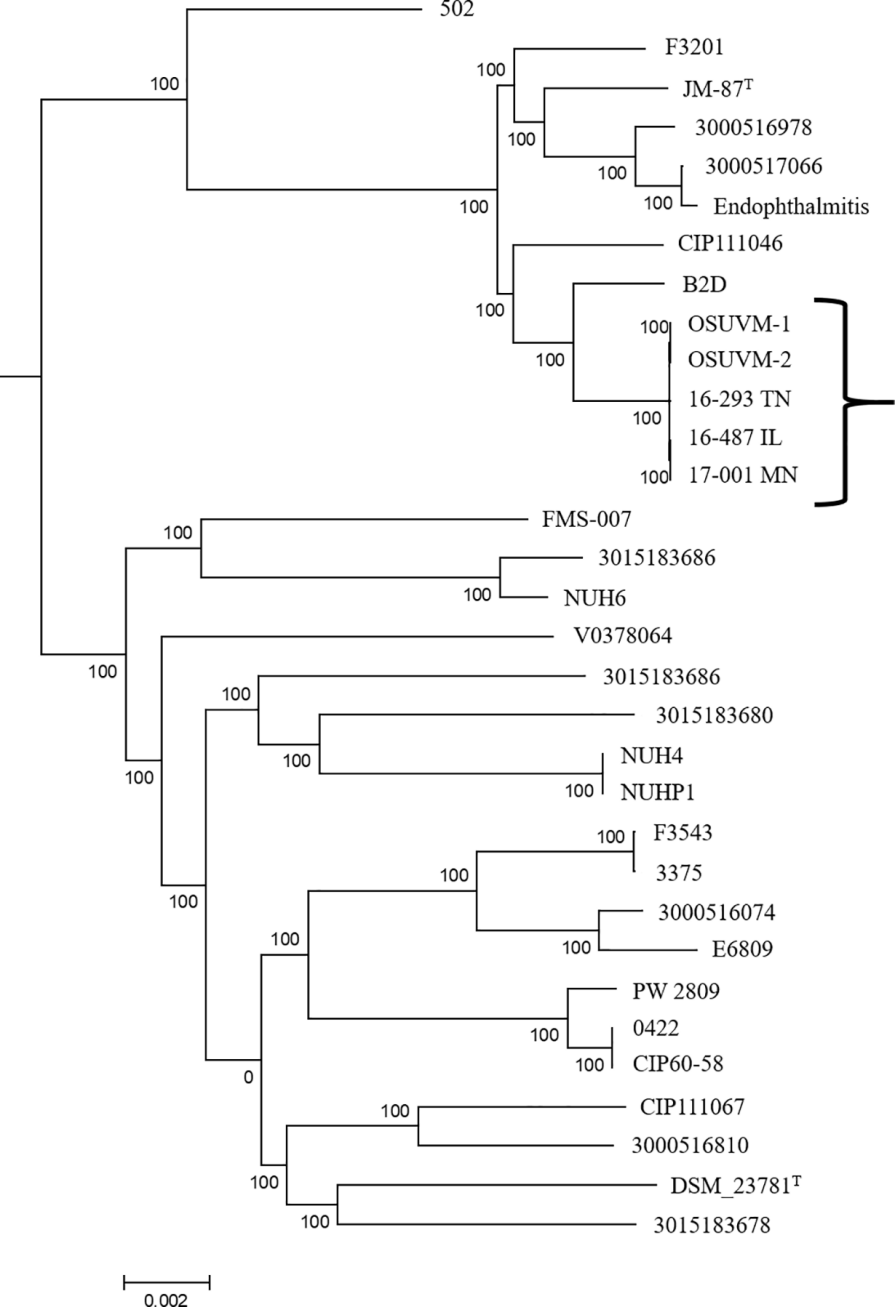
Core genome single nucleotide polymorphism tree showing the position of OSUVM-1 and OSUVM-2 compared to the *Elizabethkingia anophelis* strains reported by Nicholson *et al*. Type strains are denoted by a superscript T, and the location of the isolates from this study is denoted by a bracket.

Nucleotide BLAST and phylogenetic analysis of the core genome of both isolates revealed that both strains were *E*. *anophelis* It is of interest to note that OSUVM-1 and OSUVM-2 are part of a clade of strains resembling *E*. *anophelis* strain JM-87 [9, 74] (which was isolated from *Zea mays* stem tissue and initially described as the type strain of “*Elizabethkingia endophytica*” before whole genome sequence analysis revealed it to belonged to the *E*. *anophelis* species) rather than the clade containing *E*. *anophelis* type strain DSM_23781, which was isolated from the midgut of a mosquito (Fig 1) [9, 75].

Using the HarvestTools v1.1.2 module ParSNP, we determined that both OSUVM-1 and OSUVM-2 are closely related to *E*. *anophelis* isolates derived from human clinical specimens in Minnesota, Illinois, and Tennessee (Fig 1). A second analysis limited to OSUVM-1, OSUVM-2, and the three human clinical isolates, detected an 87% core genome among the five strains. Once ambiguous nucleotides were excluded only 198 SNP positions were located, scattered throughout the core genome of the five strains, and OSUVM-1 and OSUVM-2 differed by only 6 SNPs.

These results indicate that these five strains are highly related and that the two OSUVM isolates share commonalities with strains isolated from humans manifesting with disease caused by *Elizabethkingia*. Interestingly, Hu *et al*. [8] reported that an *Elizabethkingia miricola* strain responsible for a contagious disease resulting in black-spotted frog losses at farms in China was comparable to a human *E*. *miricola* isolate. Collectively these findings suggest that *Elizabethkingia* are not host-specific, which raises the possibility that *Elizabethkingia* might have the potential to move between humans and animals in a similar manner to known zoonotic pathogens.

### Subsystem analysis

#### Beta-lactamases

Genomic analysis of *Elizabethkingia* spp. consistently identifies multiple β-lactamases, including three characterized β-lactamases [41, 76, 77], along with a varying number of putative β-lactamases [1, 2, 4, 5, 9, 11, 25, 29, 30, 70, 78]. The 19 putative β-lactamase CDS in both OSUVM-1 and OSUVM-2 included the previously characterized class A serine β-lactamase (SBL) *bla*_*CME-1*_ [76], and metallo-β-lactamases (MBL) class B1 *bla*_*B14*_ [41] and class B3 *bla*_*GOB18*_ [77]. Of the remaining 16 putative β-lactamases, one is similar to the previously characterized class A SBL *bla*_*CIA-1*_ from *Chryseobacterium indologenes* (67% amino acid identity) [79], 11 are similar to class C SBLs, and the remaining 7 are classified as putative MBLs.

#### Multidrug efflux pumps

Efflux pumps are a key component of the intrinsic antibiotic-resistance mechanism of many bacteria and function by transporting antibiotics from within the cell to the outside [80–82]. Efflux pumps are characterized as belonging to five families: ATP-binding cassette (ABC) [83], major facilitator superfamily (MFS) [84, 85], multidrug and toxic compound extrusion (MATE) [86], resistance-nodulation-cell division (RND) [87], and small multidrug resistance (SMR) [88]. Genomic annotation of all *Elizabethkingia spp*. reveals the presence of several drug efflux pumps, yet none of these transporters has been phenotypically characterized [1, 2, 4, 5, 9, 11, 25, 29, 30, 70, 78]. RAST annotation revealed 32 CDS related to antibiotic efflux in both OSUVM-1 and OSUVM-2: 18 of the 32 CDS (56%) were identified by RAST analysis as components of RND efflux operons, 12 CDS (38%) as components of MFS operons, while the remaining 2 CDS (6%) were identified as MATE efflux pumps.

We are interested in the RND pumps in the draft genomes of OSUVM-1 and OSUVM-2 since RND efflux pumps can be a major factor contributing to clinically-relevant resistance to certain antibiotics in Gram-negative organisms [80]. Tripartite RND efflux pumps consist of an inner membrane pump attached to an outer membrane porin by way of a periplasmic adaptor protein [82, 87, 89, 90]. Although the arrangement of the genes that encode RND components varies among organisms, they can be found in a single operon in organisms such as *Pseudomonas aeruginosa* (e.g. *mexAB-oprM*) and *Campylobacter jejuni* (e.g. *cmeABC*) [87, 91]. When genes encoding the MexAB-OprM efflux pump in *P*. *aeruginosa* and the CmeABC efflux operon in *C*. *jejuni* are inactivated, a significant decrease in the MICs for various β-lactams, chloramphenicol, ciprofloxacin, erythromycin, nalidixic acid, and tetracycline is observed [90, 92–94].

The 18 CDS identified by RAST analysis as components of tripartite RND efflux pumps were all identical in OSUVM-1 and OSUVM-2 at the nucleotide level. These genes presented as six, three-gene operons, organized in the same manner as the *mexAB-oprM* and *cmeABC* operons. The OSUVM-1 and OSUVM-2 RND inner membrane pumps demonstrated 28–42% amino acid identity to MexB and CmeB, the periplasmic adaptor proteins demonstrated 24–27% amino acid identity to MexA and CmeA, while the outer membrane porins demonstrated 25–29% amino acid identity to OprM and CmeC. These homologies only suggest a relationship between these operons and characterized RND efflux systems. It should be noted that when Schindler *et al*. [95] cloned and expressed 21 genes putatively identified as encoding efflux proteins in *Staphylococcus aureus*, none resulted in increased MICs for any of the substrates tested, calling into question the function of these genes in drug efflux. As a result, it is important that the putative efflux genes from *Elizabethkingia* isolates be confirmed as drug resistance efflux pumps through biochemical analysis.

### Antimicrobial susceptibility testing

Both OSUVM-1 and OSUVM-2 demonstrated high MICs for cefazolin, ceftazidime, ceftiofur, ampicillin, penicillin, ticarcillin, ticarcillin + clavulanic acid, imipenem, amikacin, gentamicin, chloramphenicol, fusidic acid, and tetracycline (S2 Table). While the confirmed active β-lactamases in *Elizabethkingia* are known to contribute to resistance to a wide array of antibiotics that target penicillin-binding proteins [45–47], other mechanisms such as multidrug efflux, outer membrane alterations and penicillin-binding proteins that demonstrate reduced affinity for β-lactams can also contribute to β-lactam resistance, although these mechanisms remain untested in *Elizabethkingia* [81, 92, 93].

Interestingly OSUVM-1 demonstrated an oxacillin MIC of 0.25 mg/l, while OSUVM-2 showed a higher oxacillin MIC (≥ 4 mg/l), and overall OSUVM-2 displayed higher MICs for 11 of the antibiotics tested (S2 Table). Since the genes associated with resistances are identical in both strains, these MIC differences may be attributed to unidentified SNPs or specific gene content differences outside the core genome.

Both OSUVM-1 and OSUVM-2 demonstrated low MICs to ciprofloxacin and enrofloxacin, suggesting they are susceptible to these fluoroquinolones (S2 Table). Ciprofloxacin resistance in Gram-negative bacteria is driven primarily by mutations in the gene encoding the DNA gyrase A subunit (*gyrA*), and resistance is enhanced in both cases by mutations in *gyrB*, *parC*, and *parE* [96–101]. The *E*. *anophelis gyrA* encodes a predicted protein of 858 amino acids, and Perrin *et al*. [30] identified a Ser83Ile mutation in the *gyrA* of an *E*. *anophelis* strain isolated during the 2016 Wisconsin outbreak that displayed an increased ciprofloxacin MIC. Lin *et al*. [25] subsequently identified the same mutation in another *E*. *anophelis* strain which also demonstrated an elevated ciprofloxacin MIC. Thus, it is probable that the *gyrA* mutation Ser83Ile imparts ciprofloxacin resistance in *E*. *anophelis*, as it does for *E*. *coli* [102–107]. Both OSUVM-1 and OSUVM-2 contain the wild-type serine at position 83, along with two mutations, Val841Ala and Ala842Ile. Positions 841 and 842 lie outside of the region of *gyrA* thought to be responsible for fluoroquinolone resistance [96, 97, 102, 104] and the low fluoroquinolone MICs demonstrated by both strains are consistent with the expectation that these mutations would not convey fluoroquinolone resistance.

Vancomycin is used extensively for treating Gram-positive infections, in particular infections caused by methicillin-resistant *S*. *aureus* (MRSA) and *Clostridum difficile* [108, 109]. Gram-negative organisms are normally intrinsically refractory to the action of vancomycin and exhibit MICs > 64 mg/l [21], except *Elizabethkingia*, which have been reported to exhibit vancomycin MICs as low as 1 mg/l [16–19, 78, 110]. Vancomycin has been used singly or in combination therapies to treat *Elizabethkingia* infections with mixed success (reviewed in [110]). Furthermore, Hazuka *et al*. [24] reported that when an isolate of *E*. *meningoseptica* was exposed to vancomycin for 6 days, the MIC increased from 8 mg/l to 64 mg/l. Vancomycin dosing recommendations suggest that a serum trough concentration of between 15 to 20 mg/l should be reached and maintained to kill susceptible organisms, but this guidance requires that the target organism has a vancomycin MIC < 1 mg/l [108, 109, 111]. Using this standard, OSUVM-1 and OSUVM-2 (vancomycin MICs = 8 and 32 mg/l, respectively) would be resistant to vancomycin.

## Conclusion

Here we report the first two draft genomes from *Elizabethkingia* associated with horses, and that these two isolates are closely related to isolates derived from human infections, although to date no direct evidence for transmission of *Elizabethkingia* between humans and animals has been observed. We further demonstrated that both isolates display low MICs for ciprofloxacin and that both isolates display an elevated MIC for vancomycin. Clinical reports have shown potential efficacy for vancomycin in treating *Elizabethkingia* infections despite *in vitro* susceptibility results that would suggest otherwise [20, 22, 26, 32, 35, 37, 43–45], although treatment failure with vancomycin has also been reported [24, 27, 38]. We hope that this report of vancomycin-resistant *E*. *anophelis* isolates will stimulate discussion and further research to determine the efficacy (or lack thereof) of vancomycin in treating *Elizabethkingia* infections.

## Supporting information

S1 Table Distribution in coding sequence function as identified by RAST. Subsystems with differences in the number of coding sequences in the two strains are highlighted in bold.
(PDF)

S2 Table Minimum inhibitory concentrations for select antibiotics determined by the Sensititre system or broth microdilution method. Antibiotics displaying different MICs are highlighted in bold.
(PDF)
