# Supplementary material for: The draft genomes of Elizabethkingia anophelis of equine origin are genetically similar to three isolates from human clinical specimens
Source: PLoS One. 2018 Jul 19;13(7):e0200731. doi: 10.1371/journal.pone.0200731 (PMC6053191; doi:10.1371/journal.pone.0200731)
Supplement: S1 Table — Subsystems with differences in the number of coding sequences in the two strains are highlighted in bold. (PDF) [file pone.0200731.s001.pdf]

Subsystem	Coding Sequences in OSUVM-1	Coding Sequences in OSUVM-2
Cofactors, vitamins, prosthetic groups, pigments	201	201
**Cell wall and capsule**	**78**	**77**
**Virulence, disease, and defense**	**93**	**89**
Potassium metabolism	12	12
**Miscellaneous**	**28**	**27**
**Phages, prophages, and transposable elements**	**8**	**7**
**Membrane transport**	**66**	**63**
**Iron acquisition and metabolism**	**25**	**24**
RNA metabolism	121	121
Nucleosides and nucleotides	64	64
**Protein metabolism**	**203**	**225**
Cell division and cell cycle	29	29
Regulation and cell signaling	48	48
Secondary metabolism	8	8
DNA metabolism	95	95
Fatty acids, lipids, and isoprenoids	101	101
Nitrogen metabolism	12	12
Dormancy and sporulation	4	4
Respiration	66	66
**Stress response**	**70**	**71**
**Metabolism of aromatic compounds**	**19**	**18**
Amino acids and derivatives	325	325
Sulfur metabolism	15	15
Phosphorus metabolism	21	21
Carbohydrates	263	263
