# Supplementary material for: The draft genomes of Elizabethkingia anophelis of equine origin are genetically similar to three isolates from human clinical specimens
Source: PLoS One. 2018 Jul 19;13(7):e0200731. doi: 10.1371/journal.pone.0200731 (PMC6053191; doi:10.1371/journal.pone.0200731)
Supplement: S2 Table — Antibiotics displaying different MICs are highlighted in bold. (PDF) [file pone.0200731.s002.pdf]

| Antibiotic | OSUVM-1 MIC (μg/ml) | OSUVM-2 MIC (μg/ml) |
|---|---|---|
| **Amikacin** | **16** | **32** |
| Ampicillin | > 32 | > 32 |
| **Azithromycin** | **2** | **4** |
| Cefazolin | > 16 | > 16 |
| Ceftazidime | 64 | 64 |
| Ceftiofur | 4 | 4 |
| **Chloramphenicol** | **8** | **32** |
| Ciprofloxacin[a] | 0.25 | 0.25 |
| **Clarithromycin** | **≤ 1** | **4** |
| Clindamycin[a] | 1 | 1 |
| Doxycycline | ≤ 2 | ≤ 2 |
| Enrofloxacin | ≤ 0.25 | ≤ 0.25 |
| **Erythromycin** | **1** | **8** |
| Fusidic acid[a] | 16 | 16 |
| **Gentamicin** | **4** | **> 8** |
| Imipenem | > 8 | > 8 |
| **Oxacillin + 2% NaCl** | **≤ 0.25** | **> 4** |
| Penicillin | > 8 | > 8 |
| Rifampin | ≤ 1 | ≤ 1 |
| **Tetracycline** | **8** | **> 8** |
| **Ticarcillin** | **64** | **> 64** |
| Ticarcillin + clavulanic acid | 64 | 64 |
| **Trimethoprim + sulfamethoxazole** | **≤ 0.5** | **4** |
| **Vancomycin[a]** | **8** | **32** |

[a] Tested by broth macrodilution
